# Tiffany Porta Siegel (Ed.): MALDI mass spectrometry imaging: from fundamentals to spatial omics

**DOI:** 10.1007/s00216-022-04127-y

**Published:** 2022-05-25

**Authors:** Nicole Strittmatter

**Affiliations:** Fakultät für Chemie, Technische Universität München (TUM), Lichtenbergstraße 4, 85748 Garching, Germany


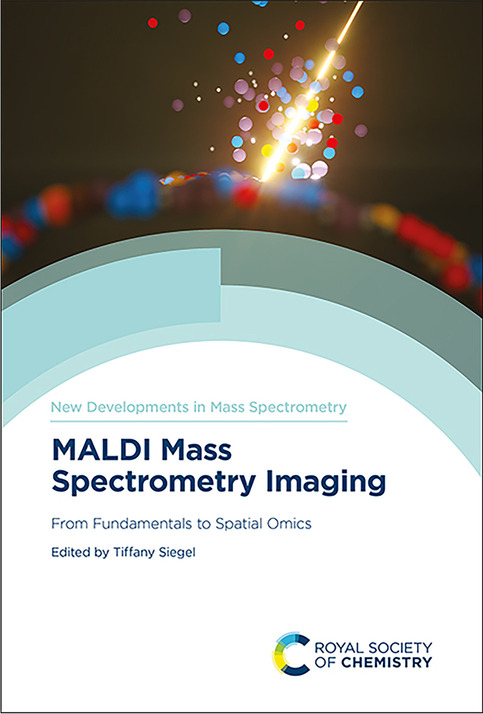


## **Bibliography**

MALDI mass spectrometry imaging: from fundamentals to spatial omics

Tiffany Porta Siegel (Ed.)

Series: New Developments in Mass Spectrometry

The Royal Society of Chemistry

ISBN: 978-1-83916-241-1

Hardcover, 500 pages,

2021

## **Book’stopic**

Mass spectrometry imaging (MSI) is a powerful technique to study the surface distribution of molecules over a wide variety of samples, ranging from food sciences to imaging of tissue specimen and plants. There has been an explosion of knowledge in the field of MSI, ranging from preparation of the sample to instrumentation and data analysis strategies. This is especially true for matrix-assisted laser-desorption ionisation (MALDI) MSI, due to its position as the most widely used imaging platform. MALDI MSI is today recognised as a mature analytical method and an essential tool in our quest to understand complex biological processes. Consequently, the pool of users is getting ever wider, currently expanding from the expert user to previously unacquainted users in medicine, food, and biotechnology. Thus, this book is a timely resource to existing and new users of this technology or those just wishing to obtain a general overview of the field.

## **Contents**

The editor explains the conception of the book into three topic-guided sections, the first covering MALDI fundamentals and implications for imaging (chapter 1–6), the second recent developments and applications in different-omics fields (chapter 7–12), and the third further application areas (chapter 13–19). Application in sections 2 and 3 are covered in a chapter each and include imaging of biological tissues, quantitative MSI approaches, microbiological applications, pharmaceutical research and development, forensic applications, environmental pollutants, clinical applications, and an additional chapter covering the analysis of eye diseases. A further chapter each is dedicated to metabolomics, lipidomics, neuropeptides, glycomics, and proteomics. The book covers all aspects from the laser, ionisation models, sample preparation, different options of image acquisition, to data analysis.

## **Comparisonwiththeexistingliterature**

Five other books are dealing with the topic of MSI in a more general fashion, *Mass spectrometry imaging* (S. S. Rubakhin, J. V. Sweedler, 2010), *Imaging mass spectrometry* (M. Setou, 2010), *Imaging mass spectrometry* (L. M. Cole, 2017), *Introduction to spatial mapping of biomolecules by imaging mass spectrometry* (B. Shrestha, 2021), and *Mass spectrometry imaging of small molecules* (Y.-J. Lee, 2022). Food applications by MSI have just been covered in *Mass spectrometry imaging in food analysis* edited by Leo M. L. Nollet, published in 2020. However, the presented book edited by Tiffany Porta Siegel, part no. 12 of the series New Developments in Mass Spectrometry published by the Royal Society of Chemistry, is indeed the first book dealing exclusively with MALDI MSI and thus allows the discussion of this topic in unprecedented detail on 500 pages (incl. references). In addition, the field has seen huge growth and improvement since its inception in the 1990s, justifying the long due arrival of a monograph on MALDI MSI.

## **Criticalassessment**

The individual chapters are written by suitable expert authors to cover the respective topic, spanning from junior to senior academics and industry experts where more appropriate as for pharmaceutical approaches, in most cases individual authors or a set of two authors. Author biographies are included in the beginning of the book. Each chapter is approximately 20 pages in length excl. references, enough to allow for drawing out an overview of the science performed within each field and guiding the reader to appropriate references for further reading. References appear at the end of each chapter in order of appearance.

The application areas discussed in this book feature predominantly biomedical imaging of tissue sections. Applications in plant, insect, or food analysis were not specifically covered; however, plants were touched upon in several chapters, including chapter 7 (metabolomics), chapter 15 (forensics), and chapter 16 (environmental pollutants). Section 2 (on ‘omics’ applications) of the book however is not restricted to sample type and further covers applications in insects and plants. Given this is a book on imaging, figures have been used sparingly and to good effect.

The book offers a concise summary of the inception of MS imaging starting from the first mention in the 1960s and developments following after that were crucial for the emergence of MALDI MSI. While giving this important look into the past, for the most part, the book delivers an up-to-date representation of the field of MALDI MSI as it stands today and a critical assessment of future challenges. It is a very well presented, clearly written, and comprehensive book, offering a good balance between theoretical descriptions of ionisation mechanisms and instrumental setups and broad practical descriptions of application areas.

## **Readershiprecommendation**

While some previous knowledge of MS and MSI will certainly improve the processing of the presented information, the book is in my opinion suitable for both the expert user and the novice in the imaging field. It is a great resource for those wishing to gain an overview over the wealth of applications and protocols of MALDI MSI but also for reading up on new application areas.

As much of the information provided in this book can be transferred to other MS imaging technologies and in absence of similar expert books on DESI MSI for instance, this book is still a valuable resource for users of other MSI technologies as it is discussing many aspects and applications with more detail than a general book on MSI can offer.

## **Summary**

In summary, *MALDI mass spectrometry imaging: from fundamentals to spatial omics* is a long overdue addition to the relevant literature and T. Porta Siegel *et al.* have made an excellent and comprehensive contribution to the MSI and MALDI MS field. I can highly recommend reading this book for prospective, new, and seasoned MSI users.

